# Untangling IGF-I signaling in the aging brain

**DOI:** 10.18632/aging.204507

**Published:** 2023-02-03

**Authors:** Jonathan Zegarra-Valdivia, Angel Nuñez, Ignacio Torres Aleman

**Affiliations:** 1 Achucarro Basque Center for Neuroscience, Leioa, Bizkaia 48940, Spain; 2 CIBERNED, Madrid, Spain; 3 Universidad Señor de Sipán, Chiclayo, Peru; 4 Department of Neurosciences, School of Medicine, UAM, Madrid, Spain; 5 IKERBASQUE Basque Science Foundation, Bilbao, Bizkaia 48009, Spain

**Keywords:** aging brain, IGF-I signaling

Decades ago, the insulin family of proteins, highly conserved along phylogeny, was established as genetic determinants of aging [1], an observation that, at that time, profoundly impacted theories of aging, and was accommodated into the former concept of antagonistic pleiotropy in aging and subsequent refinements [2]. The detrimental action on aging of insulin-like peptides (ILPs) signaling observed in invertebrate models, particularly the worm *C. elegans* and the fly *D melanogaster*, was later replicated in mammalian models, mostly in the mouse, and even included human observations [1]. While the constraints of these translational attempts from invertebrate models to mammals have been already discussed [3], mainstream thinking poses ILPs (in particular insulin and insulin-like growth factor I, or IGF-I) as detrimental in the aging process in all species, even though aging is now considered a multi-factorial and context-dependent process [2]. The latter may explain the large diversity of findings describing changes in ILP activity in the aging mammalian brain, where increased, reduced, or even unaltered ILP receptor activity have been reported in numerous publications, probably reflecting cell- and context-dependent activity of ILPs. On top of these variety of observations, to the best of our knowledge, the majority of IGF-I effects documented in the aging brain are beneficial, while the opposite seems true with the IGF-I receptor (IGF-IR). To reconcile these apparently opposing observations we postulated that aging is associated to IGF-I resistance leading to defective IGF-I functioning. We will elaborate this notion further.

Invertebrate ILPs cooperate and interact among them to carry out diverse functions such as energy allocation, sensory perception, or learning and memory. Initial observations suggest that this trend is maintained in mammals [4]. As with other double-edge processes (i.e., inflammation), insulin resistance is a physiological mechanism (i.e., sleep-associated insulin resistance) that may go awry and lead to disease, such as type 2 diabetes. Significantly, both insulin and IGF-I resistance, that are inter-related, are associated to aging; moreover, IGF-I deficiency is also associated to aging, which together with resistance, make the activity of IGF-I drastically reduced. This general loss of ILP activity along aging may argue against the concept of selection shadow (i.e., “weaker selection along aging of genes with deleterious effects on fitness” [2]) that is suggested to intervene in their purported antagonistic pleiotropy or hyper-functionality [2]. But this is a matter of discussion elsewhere. At any rate, age-associated insulin/IGF-I resistance seems maladaptive, as beneficial actions of IGF-IR ablation have been described in age-associated brain pathologies such as Alzheimer or stroke, which are both associated to IGF-I resistance. Thus, IGF-IR knock-down would ameliorate the detrimental effects of a resistant, IGF-I-independent IGF-IR.

**Figure 1:**
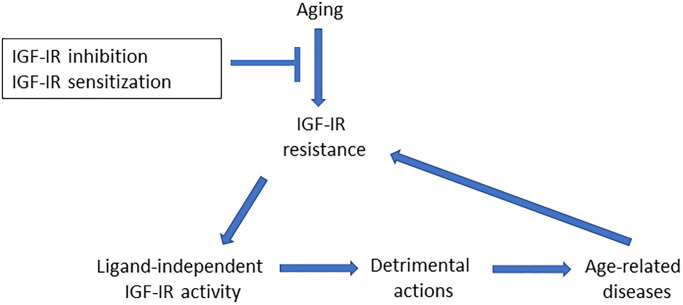
**Aging is associated to widespread IGF-IR resistance, including the brain.** As IGF-IR may act as a dependence receptor, age-related loss of regulation by IGF-I will unveil ligand-independent actions, such as pro-aging, pro-apoptosis or blockade of glucose uptake, that in turn may contribute to age-associated brain diseases. The latter may be due to unrestrained activity of the receptor since IGF-IR resistance is frequent in many age-related diseases of the brain. Conversely, age-associated brain diseases may lead to further IGF-IR resistance in a vicious cycle, as disease-associated processes such as oxidative/endoplasmic reticulum stress or inflammation elicit IGF-IR resistance. Thus, inhibition of IGF-I-resistant IGF-IR, or its sensitization to re-gain control by IGF-I, will restrain its downstream detrimental actions.

Indeed, besides the well documented pro-aging actions of IGF-IR [1], previous observations also indicated pro-apoptotic activity of IGF-IR [5], while we also recently showed that IGF-IR down-regulation in astrocytes of young mice stimulated brain glucose uptake, confirming previous observations that IGF-IR is a dependence receptor [5]; that is, its ligand-independent actions are counter-regulated by IGF-I. Thus, IGF-I, in cooperation with insulin, also stimulates brain glucose uptake by astrocytes [6]. Although somewhat counterintuitive, we suggest therefore that both pharmacological inhibition of a resistant IGF-IR or its sensitization may trigger similar neuroprotective effects (Figure 1). Specifically, the IGF-I resistant IGF-IR will exert detrimental actions in the brain in a cell- and context-dependent manner that can be prevented by either inhibiting it, or restoring its regulation by IGF-I by means of sensitizers. While this tentative explanation might seem not sufficiently founded, it is straightforward to test.

Along this vein, we recently examined the hypothalamic orexin system involved in the sleep/wake cycle as an age-sensitive target of IGF-I actions in the brain. We observed disturbed IGF-IR activity in aged orexin neurons, as determined by a combined increase in double-stained orexin/IGF-IR cells, together with decreased IGF-IR mRNA expression in these neurons, and reduced responses to IGF-I [7]. We hypothesized that, as seen in type 2 diabetes and insulin resistance, amelioration of what we interpreted as development of IGF-I resistance in aged orexin neurons, could be achieved by administration of IGF-I sensitizers. While pharmacological developments in IGF-I have been oriented towards antagonists, mostly to treat cancer, we used a newly developed IGF-I sensitizer, and observed that age-associated sleep disturbances were ameliorated, with treated old animals showing sleep patterns similar to young ones. Treatment with the IGF-I sensitizer produced comparatively better results than treatment with IGF-I, as in another set of experiments we found that diminished sensitivity to IGF-I in aged cortical cholinergic neurons was only partially restored after IGF-I therapy. This is probably because increasing the ligand does not compensate to the loss of sensitivity of IGF-IR.

Our results are apparently in sharp contrast with the reported amelioration of proteostasis after treatment with an IGF-IR antagonist in a worm model of Alzheimer´s disease (AD) amyloidosis [8]. However, if our speculation is correct, treatment of AD-like worms with an IGF-IR sensitizer would produce also a beneficial effect on proteostasis. Of note, as inhibition of IGF-IR may also lead to unwanted deleterious effects, we consider safer the use of IGF-I sensitizers. But these ideas require further study. At any rate, although the jury is still out, a nuanced understanding of the role of IGF-I signaling in the aging brain seems within reach.
